# Negotiating discord in sustainability transformations

**DOI:** 10.1073/pnas.2310186121

**Published:** 2024-04-25

**Authors:** James J. Patterson, Giuseppe Feola, Rakhyun E. Kim

**Affiliations:** ^a^Copernicus Institute of Sustainable Development, Faculty of Geosciences, Utrecht University, 3585CB Utrecht, The Netherlands

**Keywords:** transformative change, political settlements, conflict, governance, negotiation

## Abstract

Policy action for sustainability transformation faces inherent and ever-present sources of conflict, pushback, and resistance (i.e., discord). However, conceptual frameworks and policy prescriptions for sustainability transformations often reflect an undue image of accord. This involves simplified assumptions about consensus, steering, friction, discreteness, and additiveness of policy action, conferring an unrealistic view of the potential to deliberately realize transformation. Instead, negotiating discord through continuously finding partial political settlements among divided actors needs to become a key focus of policy action for sustainability transformations. Doing so can help to navigate deeply political settings through imperfect but workable steps that loosen deadlock, generate momentum for further policy action, and avoid complete derailment of transformation agendas when discord arises.

Sustainability transformations are increasingly called for in response to escalating threats to human well-being and ecological integrity. This refers to fundamental changes in society, economics, and politics (1–3)—such as through changes in institutions, norms, practices, and power relations—which bring societies toward realizing just and ecologically sustainable ways of life (4, 5). However, policy action to advance sustainability transformation faces inherent and persistent sources of discord (i.e., conflict, pushback, and resistance). Discord arises from interlinked material (e.g., interests, resources, authority) and interpretive (e.g., problem frames, beliefs, values) struggles. Political parties and incumbents often oppose policy action when their interests or values are at stake. Individuals might reject policies that they perceive as overreach, especially in polarized settings (6, 7). Unsustainable systems can be “locked-in” from multiple angles (8) leading to unintended or unforeseen consequences from policy action (9, 10). External shocks can also undermine support for action, such as by delaying fossil fuel phase-outs due to energy crisis (11). The challenge of discord calls into question the adequacy of existing conceptions of policy action for sustainability transformation as reflected in many prominent frameworks and prescriptions.

Although most scholars and policymakers recognize the potential for discord in sustainability transformations, conceptual frameworks, and policy prescriptions often tend to underestimate its significance and centrality. Discord is often treated as an aberration that can be overcome through consensus-building, political will, or win–win solutions. Ecological imperatives are given precedence over political considerations, or politics is perceived as less decisive than objective earth system processes. This can lead to an emphasis on technically derived sustainability targets and roadmaps that are assumed to be universally desirable and ex ante achievable. However, real-world politics rarely conforms to the assumptions of scientific plans and targets. Sustainability transformations are “peopled” rather than instrumental processes (1, 12, 13) and political differences among actors are often persistent or even irreconcilable (14), challenges long recognized by scholars concerned with policy action in fractious and wicked settings (10, 15–19). Transformation might require reorganization of power structures and redistribution of resources (20) leading to winners and losers (21) and upending extant cultural norms and values. Yet, the emphasis on accord within prominent frameworks and prescriptions informing debates on sustainability transformation leaves us ill-prepared to address real-world discord involved in advancing policy action. Policy action must navigate divergent interests and moral stances among heterogeneous actors and complex and messy processes of societal change.

Advancing policy action for sustainability transformation requires recognizing that accord is not necessarily a realistic prerequisite for transformation and instead the focus needs to be on finding ways to pragmatically negotiate discord. This entails moving away from a logic whereby agreement is sought for predetermined solutions (an “accord-based view”) to a logic whereby discord is recognized as the basic condition within which policy action is pursued (a “discord-based view”) (Fig. 1). Continuous negotiation among deeply divided actors becomes central to understanding how policy action can be generated within fractious sociopolitical settings rather than seen as something that can be imposed from outside (22–24). Building on the rich literature on the dynamics of sustainability transformations, policy action in fractious and wicked settings, and approaches to navigating conflict in peace and conflict studies, we offer a fresh perspective on how to harness and negotiate discord in sustainability transformations.

**Fig. 1.**
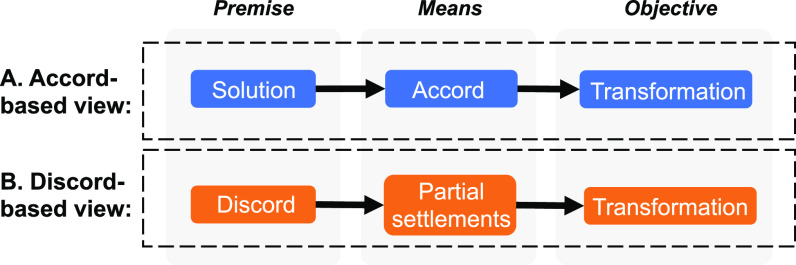
Two views on action for sustainability transformation: (*A*) Accord-based view where solutions are first identified and then accord is obtained from all involved actors, or (*B*) discord-based view where discord is taken as the starting point requiring ongoing efforts to (continuously) find partial political settlements among limited sets of actors.

We argue that such negotiation must involve continuously finding “partial political settlements”: Uncomfortable compromise among deeply divided actors that helps to realize a minimal degree of productive cooperation in a situation that otherwise risks intractable conflict and failed transformation. This differs from conventional approaches to negotiation focused on joint gain which might not be possible in situations of discord and instead requires finding temporary truce among intractable interests and moral stances. The benefit is a focus on generating workable steps that potentially help to loosen deadlock and avoid complete derailment of transformation agendas. Partial political settlements may be imperfect (e.g., considering scientific targets), tentative, and susceptible to reversal, but they can help to shift political conditions and make new actions possible. This is not about limiting ambition, but rather, finding a way to conceive of endogenous policy action that has the potential for transformative effects given inevitable discord, even if this is not guaranteed.

## Policy Action for Sustainability Transformation

### Common Frameworks and Prescriptions.

Frameworks and prescriptions for sustainability transformations frequently underrecognize discord. This occurs when the goal of transformation is assumed to be universally shared and desirable, pathways are portrayed as mappable by backcasting from future goals, and processes of societal transformation are seen as controllable through intervention and steering. For example, recent proposals for integrated pathways for global transformation (25), mission-oriented innovation policy (26), and a social contract for sustainability (27) emphasize collaboration and investment around presumed shared priorities that may not actually exist. Roadmaps for decarbonization (28) can suggest politically implausible sequences of action that overlook adverse impacts (29) and counterclaims among local communities (30). Conceptual frameworks may depict societal change as a transition from one stable configuration to another (31), pathways that can be controlled at a global scale (32), or social tipping of new practices that propagate through society (33, 34). However, societal change is inherently unstable and contingent. There is no singular vantage point from which to steer transformation especially on a planetary level, and the spread of new practices is often constrained, uncertain, and contested.

Empirically, we observe the occurrence of discord in response to policy action in various ways (Table 1). For instance, policies aimed at promoting clean energy transitions in wealthier countries may encounter resistance from local communities elsewhere who bear the burden of environmental and social harms that are externalized. Despite policy action to encourage renewable energy, incumbents may push back against change. Generating and sustaining support for phasing out fossil fuel production is often challenging especially in changing societal and geopolitical contexts. Additionally, policy actions may be perceived differently by individuals even within a single territory (such as a city), due to peoples’ diverging preferences and historical experiences. Governments themselves may undermine their own environmental protection laws, and people may resist behavioral change demands they deem unrealistic in their everyday lives. Consequently, policy action for sustainability transformation is far from unproblematic and long-term durability may be lacking. Moreover, the potential for discord might also increase as policy action becomes more demanding.

**Table 1.** Examples of discord arising in policy action for sustainability transformation

Policy action	Sources of discord	References
“Green Deal” policies for climate neutrality in wealthier countries (e.g., electric vehicles) create demand for materials.	• People in places where materials are sourced or disposed resist bearing externalized environmental and social harms (e.g., minerals extraction, waste).	(29, 35, 36)
Energy policies for upscaling renewables (e.g., R&D funding, incentives) without removing support for fossil fuels.	• Unsustainable technologies compete with renewable technologies. • Incumbents retain power to stymie new renewable developments.	(37–39)
Fossil fuel phase-out policies or agreements (e.g., coal mining).	• Consensus for phase-out can be difficult to obtain and sustain, especially under external shocks and political competition.	(11, 40)
Smart cities policies for resource use efficiency and monitoring.	• Use of technology can be resisted especially when perceived as untransparent, undemocratic, and/or lacking attention to historical inequalities.	(41–43)
National biodiversity conservation laws.	• Governments may undermine their own biodiversity conservation laws in a range of ways in response to domestic and international pressures over time.	(44)
Instrumental behavior change interventions (e.g., mobility, energy use, consumption) implemented in isolation of context.	• People may resist behavior change expectations if they conflict with everyday practices, norms, and infrastructures. • Resistance may be especially strong if behavior change requires high wealth.	(45–47)

### Assumptions about Transformation Dynamics.

**Fig. 2.**
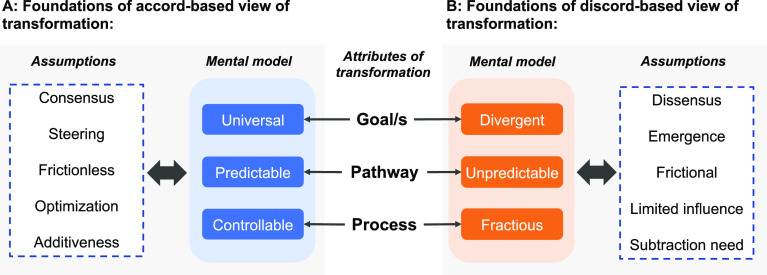
Mental models and associated assumptions about sustainability transformations underlying (*A*) accord-based view and (*B*) discord-based view, which underpin frameworks and prescriptions informing policy action.

Insufficient attention to discord within existing frameworks and prescriptions is related to the implicit mental models of transformation invoked. Attributes of transformations such as goals, pathways, and processes of change can be conceptualized in differing ways (Fig. 2). One approach is to adopt a mental model of transformation that assumes universally shared goals, mappable pathways, and controllable processes of societal change. This forms the basis of what we term an “accord-view.” Another approach is to adopt a mental model of transformations that acknowledges inherently divergent goals, unpredictable pathways that only become apparent in hindsight, and fractious processes of societal change. This underpins what we term a “discord-view.” While the former view may serve as a useful heuristic to inform policy action, particularly when there is a sense of urgency regarding sustainability issues, its practical applicability and effectiveness should not be presumed. The latter view is closer to reality and therefore essential as a starting point for policy action in real-world contexts.

However, despite the centrality of discord, an accord-based view continues to dominate debates about how sustainability transformations may be realized. This perspective often assumes the potential for consensus, the capacity to steer and control processes of change, the absence of decisive friction or conflict, the ability to optimize policy action, and the adequacy of additive actions without also dismantling unsustainable elements (Table 2). From the standpoint of a discord-based view, these assumptions reflect biases toward an overly optimistic portrayal of accord. Not all conceptual frameworks and policy prescriptions necessarily embody all of these biases. However, the coexistence of these biases is common, leading to a systematic deviation from the realities of discord in approaches and debates on sustainability transformations.

**Table 2.** Common biases in an accord-based view of sustainability transformation

Bias	Description	Policy manifestation	Alternative
Consensus	Ex ante consensus on transformation goals can be reached among diverse actors.	Belief that widespread agreement on specific policy action is possible.	Pervasive dissensus.
Steering	Transformation can be steered or controlled to achieve pre-defined outcomes.	Emphasis on systemic planning to develop comprehensive pathways.	Transformations are emergent, and unpredictable.
Frictionlessness	Transformation is unencumbered by pushback, or that this can be mitigated.	Overlooking or downplaying resistance and the possibility of backfires.	Persistent and multifaceted friction is inevitable.
Discreteness	Transformation can be achieved through specific well-targeted policy actions.	Attempts to find optimal policies or leverage points with outsized impact.	Instrumental prioritization is no guarantee of impact.
Additiveness	Actions can be added to an existing system without attention to subtraction.	Policies for innovation and/or upscaling without also dismantling unsustainable elements.	Need for both additive and subtractive action.

A consensus bias is evident in appeals to a common “we,” which, in reality, is a myth in heterogeneous societies. This bias can be observed in documents like “The Future We Want” (48), where, contrary to what is implicated, the “we” refers only to heads of states and governmental representatives. The implementation of the 17 Sustainable Development Goals (SDGs) has been uneven, with individual states selectively prioritizing them (49), further highlighting the absence of a common “we.” The aspiration for an overarching goal for the SDGs (50) did not materialize for the same reason. The bias toward consensus is also reflected in the expectation that collaboration can reconcile competing interests, moral stances, and power imbalances among actors. However, collaboration and stakeholder participation are not detached from existing social dynamics and power structures. They can perpetuate power imbalances and lock participants into nonnegotiable positions (51). While dialogue is important, disagreements and diverging values (dissensus) are widespread (13), making it challenging to reach a consensus on a “right” course of action. Moreover, any consensus achieved may be temporary and susceptible to erosion due to external shocks such as energy or food price spikes or threats to incumbent interests as policy actions ramp up (37).

A steering bias is evident in pronouncements about the ability or necessity to steer overall societal systems toward a particular “direction” (32, 52). This bias can overstate the level of control that policymakers and other actors have over complex problems (14, 53–55). While steering is often presented as well-intentioned, its feasibility and adverse consequences are often neglected. Relatedly, efforts to comprehensively map and manage complex systems (56) or pathways (57) can also confer a misleading sense of control. Although such knowledge is valuable, implied policy action is not necessarily feasible or unproblematic. Transformative pathways cannot be predetermined and strictly followed. Instead, they emerge from a multitude of policy actions that are inherently limited in their scope, occurring across various contexts and unfolding over time in unpredictable ways (14, 58, 59). Balancing attempts to steer outcomes while allowing for emergent societal responses is undeniably challenging, especially considering the urgent warnings about biophysical thresholds (60, 61).

A frictionlessness bias is evident in policies and frameworks that underestimate or downplay the potential for resistance (1). Although few would disagree that diffuse resistance to policy action exists, it is often treated as merely an obstacle to be overcome rather than a fundamental aspect of societal change. For example, frameworks of social tipping points assume that decision-makers may actively trigger a critical threshold (33, 62) after which new practices and values will spread throughout society (34). However, resistance is not solely a matter of individual behavior but also emerges from complex relationships with technologies, institutions, and broader cultural contexts (8, 63). Furthermore, resistance is increasingly observed in various domains, including opposition to renewable energy infrastructure (30, 64), climate adaptation initiatives (65, 66), and climate mitigation policies (67). Resistance is not merely a consequence of transformation but also a constitutive part of its dynamics (68, 69). While the specific forms of resistance may not always be predictable, the potential for many sources of resistance should be expected.

A discreteness bias is evident in efforts to prescribe targeted interventions that are believed to have outsized potential for impact. The notion of leverage points, where interventions can be strategically applied to gain deeper traction (70, 71) and policy mixes that combine multiple policies to overcome limitations of individual policies (72) reflect a desire for efficiently targeted policy action. While these approaches can be valuable, the emphasis on broad diagnostic heuristics may create a misleading perception of the actual capacity of real-world actors to intervene at the specified location or level and to realize the intended impacts (55). For example, identifying leverage points heuristically does not guarantee the feasibility of taking action at those points or ensure that the desired effects will be realized (70). Similarly, the belief in singular interventions, such as charismatic leaders, social mobilization, exemplary cases, or specific policies, as triggers for transformation oversimplifies the complexity of these phenomena and overestimates their potential impact. Such thinking can even reflect a tendency toward panaceas, where a single solution is seen as a cure for complex problems (73).

Last, an additive bias is evident in policy discussions that emphasize innovation and scaling as the primary means of realizing transformation (74–76). This perspective can create the perception of win–win solutions that overlooks the challenging task of dismantling unsustainable aspects of existing systems, such as infrastructure, policies, and political economic arrangements (55, 77). In the context of decarbonizing industrial economies, it is necessary to phase out fossil fuel technologies, subsidies, and consumption practices. This requires dismantling existing barriers, both legal and infrastructural, to make way for low-carbon alternatives (38, 39, 78). Initiatives like the Fossil Fuel Non-Proliferation Treaty and campaigns advocating for the removal of fossil fuel subsidies and divestment from fossil fuel industries recognize the need for subtractive actions (79, 80). Such subtractive actions involve confronting established power relations and explicitly acknowledging the political economic gains and losses for different actors, sectors, and communities.

### The Challenge of Discord.

Discord is an important starting point for policy action on sustainability transformations. We should expect that dissensus is pervasive, the capacity to steer system change is limited, friction is inevitable and ubiquitous, impact can rarely be levered in a straightforward way, and additive and subtractive action are intertwined (Table 2). Many scholars would readily affirm these challenges. However, there remains a lack of conceptual frameworks and pragmatic approaches to policy action that directly address discord in sustainability transformations. This is especially apparent under a paradigm of governance for sustainability focusing on choices and action rather than rules and institutions (81). Although problems may be understood as complex and wicked, the political behavior of actors is crucial to both producing and navigating such intractability (10, 82, 83), foregrounding the political challenges of policy action for sustainability transformation.

Policy action faces complex situations that involve not only actors with competing interests, but also with divergent moral stances regarding a particular course of action, such as different perceptions of justice, freedom, and democracy. For example, the Yellow Vests protesters in France in 2018 to 2019 contested a fuel tax increase implemented as part of climate policy, arguing that it unfairly burdened individuals living outside urban areas who were already struggling financially (84, 85). Similarly, nationwide farmer protests in the Netherlands from 2019 onward, in response to proposed measures to reduce nitrogen pollution in agriculture, reflected concerns about perceived undemocratic policy overreach in rural regions (86). In broader discussions related to the 2030 Agenda for Sustainable Development, developing countries rejected the concept of planetary boundaries, as they believed it prioritized global environmental concerns over local social needs (87). On an everyday level, climate policy measures can face criticism when they are perceived to limit personal freedom, such as in relation to consumption choices (88) and mobility (89). These types of situations exemplify the coexistence of what Margalit (90) describes as an “economic” view of politics, which is open to bargaining for joint gain, and a “religious” view of politics concerned with safeguarding values and ways of life.

When facing deep divisions among actors, approaches to policy action are needed that acknowledge and address discord as the basic starting condition. One option is to integrate a recognition of discord into an accord-based view by placing greater emphasis on social acceptability, pluralism, and opportunities for deliberation. While this may be appropriate in some situations, it remains limited by assuming the possibility for shared goals, collaboration, and responsiveness to information. Arguably, any approach too strongly premised on a “common good” will be unrealistic. Another option, building on the observation that incremental or transformative change is too stark a dichotomy (3, 91), could be to pursue “small wins” which are suggested to be less likely to trigger resistance than radical intervention (92). However, such an approach still tends to be premised on a shared goal, and resistance may not be proportional to the scale of intervention since seemingly successful actions may highly contingent and scales of possible action might vary in different moments or venues. Related but distinct is the idea of “clumsy solutions” which combine elements reflecting different cultural beliefs about society to appeal to heterogeneous communities within a “plural and argumentative system” (16, p. 828). A third option could be to pursue agonistic politics which argues that conflict should be embraced as an inherent aspect of politics, and societies should strive to keep democratic political struggle permanently open (93). However, this offers limited practical guidance for policy action and does not clearly address the challenge of incorporating time-sensitive imperatives, such as environmental crises. Hence, what is needed is approaches that focus on negotiating discord in ongoing processes of societal transformation toward sustainability. This builds on observations in policy studies (10, 15, 16, 82, 83) and peacebuilding and conflict studies (23, 24, 94, 95) that responses to conflict may inevitably be partial and provisional, occur across multiple arenas in society within and beyond the state, and require ongoing and often uncomfortable political work.

## Negotiating Discord through Partial Political Settlements

We posit that negotiating discord requires continuously finding partial political settlements among deeply divided actors. The notion of a partial political settlement refers to a negotiated but uncomfortable compromise to realize a minimal degree of productive cooperation among key actors who influence a policy outcome in a situation that otherwise risks intractable conflict and failed transformation. We derive this from the broader notion of a political settlement within postconflict transitions, where the emphasis lies on the cessation of hostilities among rival actors to allow new collectively beneficial developments to emerge and consolidate. While differing views exist in the literature, we adopt a conception of settlements as deliberate agreements (94–96), in line with our focus on deliberate policy action. The notion of political settlements can inspire thinking about how deep or intractable conflict can be negotiated in an at least minimally sufficient way to enable future collective benefits to accrue. Such efforts would always be incomplete and require ongoing work through the dispersed activities of many different actors in multiple spaces, both within and beyond the formal political arena. Thus, any particular settlement will be “partial,” and multiple settlements will be needed to forge a pathway toward sustainability transformation.

Negotiating partial political settlements is challenging due to the presence of both competing interests and diverging moral stances. Negotiation is typically thought of as involving bargaining among competing interests to secure joint gain. While rarely straightforward, this is often resolvable in principle through sharing of costs and benefits or compensation to policy losers (97). However, negotiation becomes more difficult when actors hold diverging moral stances, especially in the context of entrenched political ideologies, weakened shared norms, and social polarization. Hence, both bargaining between competing interests as well as bearing the weight of diverging moral stances are needed. This is precisely where the notion of settlements is helpful.

**Fig. 3.**
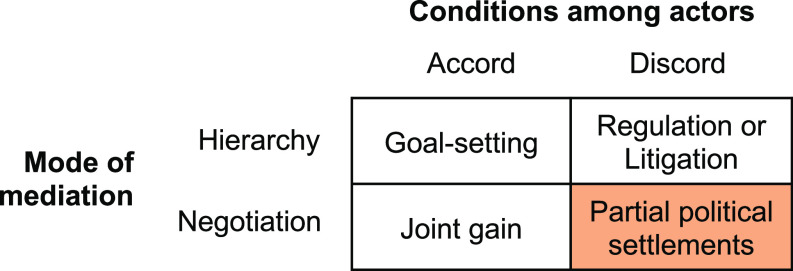
Approaches to policy action under different circumstances, where partial political settlements involve negotiation in the presence of discord.

Partial political settlements represent something that actors can live with rather than something that they necessarily fully embrace, and as such, may not fully feel like joint gain to those involved. Rather than seeking to find an elusive common good, partial political settlements focus on finding a least-bad or somewhat-common good, downplaying the provision of definitive solutions (24). Thereby, this approach engages with discord by seeking to internalize mutual discomfort rather than avoiding it, which can help to shift political conditions and make new actions possible. It also recognizes that future action will continue to encounter discord since politics is ongoing, and action may not propagate throughout society. The relation between action and effects will be nonlinear; transformative outcomes might not come from a single settlement but rather from multiple settlements unfolding in different arenas over time. Altogether, this offers a pragmatic orientation for policy action in situations where discord is inevitable, instead of an unrealistic pursuit of joint gain especially when hierarchical authority is not readily available to mediate dispute (e.g., due to political deadlock and/or counter-opposed power among multiple actors) (Fig. 3).

Placing emphasis on partial political settlements could enable new forms of policy action through unconventional coalitions. For example, compromises reached among environmentalists, industry representatives, or utility incumbents could pave the way for the adoption and uptake of innovative policy measures (98–100). This could foster the development of different social norms centered around (possibly uncomfortable) compromise by showcasing viable instances of cooperation in situations where it appeared impossible, initiating feedback toward reinforcing a new policy trajectory. However, it is possible that not all settlements will lead to productive outcomes, and may even result in diluted or co-opted agreements that fail to generate broader impacts or inspiration (101). Peacebuilding and conflict studies scholars observe that sometimes settlements contain conflict but do not address its underlying causes (23, 94). The difference will depend on whether a particular settlement expands or restricts opportunities and capabilities for further action. For example, the effects of settlements could include stimulating different coalitions (102), building different state capacities and/or shifting societal expectations (103), and empowering forms of autonomous action beyond the state (104). But evaluating the success of a partial settlement will also not be solely objective (e.g., extent of rule change, robustness of support) and will also depend on narrative interpretations promoted by various actors (105) regarding the benefits of the settlement and its long-term role in a transformative pathway.

Policy action aimed at finding partial political settlements will inevitably vary across different issues, venues, and contexts. It may even require governance structures that do not yet exist on a widespread scale. An example is the introduction of coal phase-out commissions in Germany and Canada, which served as unique platforms bringing together diverse stakeholders such as government officials, industry representatives, workers, and citizens to develop workable strategies for transition (40). Both cases involved extensive multiactor negotiations to address challenging decisions regarding policy action for societal transformation, starting from a position of disagreement and tension. In the German case, despite ongoing dissatisfaction among certain stakeholders, it appears unlikely that the phase-out decision will be reversed (106), even with delay pressures due to the subsequent energy crisis. Another example of a partial political settlement is the negotiation of climate provisions in the Inflation Reduction Act of 2022 in the United States, which entailed compromises among deeply divided political actors. This compromise has created favorable conditions for the emergence of energy transition industries and supportive alliances (107), opening up the possibility for further policy action down the track that may not have been achievable otherwise, thanks to investments in renewable energy and market development. However, as an approach primarily focused on incentives, additional targeted efforts may be required to address the dismantling of fossil fuel systems.

Issues such as power differences, marginalization, and exclusion can undermine partial settlements. Sustainability scholars warn about elite capture of processes, exclusion of groups who are already marginalized, and reproduction of existing inequalities (1, 108–110). In response, they highlight the need for inclusion, attention to power relations, and to embrace plural forms of knowledge and experience in ongoing struggles over societal transformations (14, 21, 108, 111). At the same time, peace and conflict scholars observe that comprehensive inclusion can be hard to realize (23, 112), and may sometimes require working in more limited ways (94) or even with actors deemed undesirable (22) for a broader outcome. They suggest strategies including support for marginalized groups to sustain the capacity for contestation, mobilization of broader values to motivate action beyond particular interests, and use of mediation to enable endogenous actors themselves to identify problems and possible responses, including trusted actors who can bridge divided groups (such as faith-based, business, trade union, or other civil society groups) (22, 23, 94, 95). These strategies could be beneficial for supporting the emergence of partial political settlements in sustainability transformations too. Importantly however, there will be “no simple solutions” to issues of inclusion and democracy (108), and any individual settlement will be, by definition, nonideal. However, this very character is what enables them to remain open to both questioning and innovation.

## Conclusions

In this Perspective, we offer a provocation to rethink approaches to policy action for sustainability transformations recognizing the basic starting condition of discord. This is important for moving beyond widespread restatements and longstanding pleas for consensus, political will, and win–win solutions, which are often unrealistic and elusive in real-world transformation processes, especially as the demands for policy action increase yet become more contested. This is not to say that continued efforts to realize accord are not needed, but that alongside (and in conversation) with this, more attention is needed to find ways to engage with the challenges posed by inevitable discord.

We propose the concept of partial political settlements as a pragmatic lens for policy action that embraces the discordant nature of societal change and transformation, rather than relying on an overly optimistic view of achieving accord. Partial political settlements are imperfect and tentative, requiring ongoing work rather than being treated as one-time solutions. This aligns with the need for attention to processes of governance for sustainability (81). Crucially, our approach focuses attention on the endogenous development of policy action for sustainability transformation within a society, rather than envisioning policy action as imposed from an external position. It also encourages different ways of thinking about policy action, moving beyond more-of-the-same prescriptions such as more stakeholder participation or political will. Nonetheless, pursuit of partial settlements provides no guarantee of success in realizing transformative change at the pace and scale understood scientifically to be needed. But neither does a focus on idealized notions of policy action through accord. Importantly, our argument does not imply that discord makes substantial political action, and hence transformation, impossible. Rather, it represents a pragmatic approach that tries to take discord seriously and learn from other fields which also confront apparently intractable discord and conflict, to help move beyond an image of transformation as universal, predictable, and controllable.

Pursuing partial political settlements may require different governance processes and arrangements. This could include platforms or mechanisms that strategically mediate between opposing actors. The specific nature of such arrangements will vary across contexts, depending on factors such as legal rules, past experiences, political opportunities, and social preferences. Determining what qualifies as a genuine settlement (rather than, for example, co-optation or delay) may be ambiguous and involve interpretation of what is meaningful in a specific time and place (24) and thus identifying the circumstances under which partial political settlements have transformative effects is an important research need. Understanding network effects (e.g., interaction, spillover, cumulation) and temporal consequences (e.g., feedbacks, norms, new possibilities for action or cul-de-sacs) of settlements, as well as the roles of different actors in different arenas become further key priorities. Future research might also usefully investigate how partial political settlements interact with other forms of action (e.g., protests, lobbying, participation, goal-setting) both within and beyond formal political processes. At the same time, insights will also come from empirical cases that confront discord and experiment with ways to deal with it. Scholars should therefore also watch closely and learn from practice in this regard.

## Data Availability

All study data are included in the main text.
